# A proposed consensus panel of organisms for determining evolutionary conservation of mt-tRNA point mutations

**DOI:** 10.1016/j.mito.2012.06.009

**Published:** 2012-09

**Authors:** John W. Yarham, Robert McFarland, Robert W. Taylor, Joanna L. Elson

**Affiliations:** a Mitochondrial Research Group, Institute for Ageing and Health, The Medical School, Newcastle University, Newcastle upon Tyne, NE2 4HH, UK; b Department of Statistical Genetics, Institute of Genetic Medicine, Centre for Life, Central Parkway, Newcastle University, Newcastle upon Tyne, NE1 3BZ, UK

**Keywords:** Mitochondrial DNA disease, mt-tRNA variation, Evolutionary conservation, Pathogenicity, Scoring system, Intra-species variation

## Abstract

Assigning pathogenicity to mt-tRNA variants requires multiple strands of evidence. Evolutionary conservation is often considered mandatory, but lack of a standard panel of organisms to assess conservation complicates comparison between reports and undermines the value of conservation-based evidence. We demonstrate that intra-species *MTT* sequence variation is sufficiently low for sequence data from a single organism to adequately represent a species. On this basis, we propose a standardised panel of organisms for conservation assessment and describe integration of this conservation panel into a pathogenicity scoring system designed to assess mt-tRNA variation associated with mitochondrial disease.

## Highlights

► Assigning pathogenicity to mt-tRNA variants requires multiple veins of evidence, including evolutionary conservation; however, there is no “standard sequence panel”. ► Species used for conservation assessment previously, noting alarming variation. ► Whether a panel with a single sequence from each organism can be used to assess conservation, given intra species variation. ► We set out robust criteria for sequence selection and suggest a “standard sequence panel”. ► Aid decisions on when to carry out functional studies upon identification of novel mutations and improve consistency of mt-tRNA mutation reporting.

## 1 Introduction

Mitochondrial (mt-) diseases caused by mutations in genes of both mitochondrial (mtDNA) and nuclear (nDNA) DNA affect both adults and children, and are both clinically and genetically heterogeneous. Characteristic symptoms of these often multi-system disorders include epilepsy, myopathy, deafness and ophthalmoplegia, with point mutations of mtDNA, particularly in the 22 mt-tRNAs being increasingly recognised as important causes of disease (Florentz et al., 2003). Determining whether an identified variant is disease-causing (i.e. pathogenic) or not (i.e. polymorphic) remains challenging, due to complications arising from interactions with the nuclear background, tissue segregation and the threshold effect as well as the lack of a consistently observed phenotype:genotype correlation (Yarham et al., 2010).

Recently, pathogenicity scoring systems have been designed for both mt-tRNA point mutations (Yarham et al., 2011) and point mutations within the mtDNA-encoded Complex I genes (Mitchell et al., 2006), in order to classify identified variants associated with disease more accurately. These scoring systems classify the likelihood that a variant is pathogenic using evidence from multiple sources, based upon the canonical criteria of pathogenicity established by DiMauro and Schon (2001). The scoring systems integrate the elements of these original criteria with additional evidence including histochemical analyses and functional studies.

Far from being peripheral, evolutionary conservation is cited in support of pathogenicity in as many as 80% of reported mutations. However, an increasing number of pathogenic mt-tRNA mutations are being identified at sites that are not well conserved (e.g. m.4284G>A (Corona et al., 2002), m.7497G>A (Jaksch et al., 1998) and m.8344A>G (Shoffner et al., 1990)), suggesting that evolutionary conservation alone cannot be used to assign pathogenicity (McFarland et al., 2004). Today, functional studies that include *trans*mitochondrial cybrid, mt-tRNA steady-state level and single fibre studies are considered the gold-standard. They are essential for assigning pathogenicity to mt-tRNA mutations, whilst data from other criteria provide additional supporting evidence, sometimes used to determine whether functional studies should be conducted (Yarham et al., 2011).

The lack of an agreed panel of species that can be used for assessing conservation means that currently, a research paper can report “evolutionary conservation” using an arbitrary selection of organisms. Using conservation data inconsistently is counter-productive and might be misleading as there is the potential for species to be “cherry-picked”. For evolutionary conservation to be of greatest utility, it is essential that the variability in species selection between studies be addressed so that consistent statements regarding the extent of conservation can be made. Two main issues must be tackled; firstly, whether a single sequence is sufficient to reliably represent the species, given the likelihood of considerable intra-species variation, as seen between human haplogroups (Pereira et al., 2009); secondly, which organisms should be included in a standardised consensus conservation panel?

We hope that the panel of organisms proposed will be an aid to the consistent reporting of conservation of mt-tRNA variants, with the aim of improving the reliability of pathogenicity assignment, and facilitating comparison between different reports. Given the role that evolutionary conservation plays in our pathogenicity scoring system, we also outline the manner in which variants should be scored with regard to the conservation of the affected position.

## 2 Methods

### 2.1 Frequency of species reporting

A previously performed literature review (Yarham et al., 2011) was extended up to and including 31st September 2011 by again searching PubMed (http://www.ncbi.nlm.nih.gov/pubmed) with the terms “mitochondrial”, “tRNA” and “mutation”, to identify all reports of mt-tRNA variants in association with disease irrespective of their resulting pathogenicity classification. A total of 369 publications have reported 214 different mt-tRNA variants in association with disease. Of these publications, 120 describe a defined panel of organisms that were used to assess conservation, and these were used to calculate how many times each species has been utilised. The other 249 publications either reference previous work on the variant of interest, make no comments regarding conservation or reference a database such as Mamit-tRNA (http://mamit-trna.u-strasbg.fr/) (Putz et al., 2007) or tRNAdb (http://trnadb.bioinf.uni-leipzig.de/) (Jühling et al., 2009).

**Supplementary Fig. 1:**
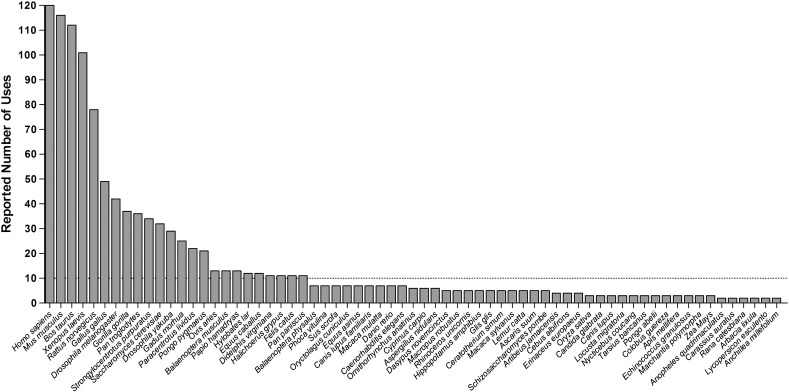
Reported utilisation of species for conservation assessment. This graph illustrates the range of species used to determine conservation when reporting mt-tRNA mutations. All species used twice or more are shown and clear demarcations between species used more than 50 times, more than 20 times and more than 10 times can be seen. Our threshold of 10 or more reports is indicated by the dotted line, and ensures that only the most commonly used species were considered for inclusion in the panel, whilst keeping the range of possible species as broad as possible.

Species that had been included in fewer than 10 conservation panels were not considered for further analysis. The logic for this inclusion threshold was that only the most popular species would be considered for the consensus panel, and that idiosyncratic reporting of particular species by a single research group would be excluded. An overview of the reported usage of species in conservation panels (Supplementary Fig. 1) supports this limitation, with a clear demarcation between those species reported on 7 and 11 occasions respectively. There is a more marked distinction between those species reported on 13 and 21 occasions, but this significantly limits the number of species that could be used in the panel.

### 2.2 Assessing intra-species variation

A search of the GenBank® database (GenBank ID: BA123456) (Benson et al., 2011) was performed (on 10th October 2011) using the search term “mitochondrion genome *species latin name*”. All available mtDNA genome sequences for each of the species utilised 10 times or more were identified. All incomplete genomes, as well as sequences generated from cell lines or clones were excluded and the number of sequences available were counted. For those species with in excess of 34 complete mtDNA genomes listed in GenBank®, we utilised a random number generator to select 34 sequences for comparison with the reference sequence. This cut-off was employed arbitrarily for the purposes of consistency, given that the majority of species had ≤ 35 sequences whilst the few species with > 35 sequences had in most cases several hundreds available. Using all of these sequence data would therefore be of little benefit given the relative paucity of data for most of the species. If fewer than 34 complete mtDNA genomes were available, partial sequences were also randomly selected for inclusion, up to a total of 34 sequences. Only species with multiple sequences available in GenBank® were considered for inclusion in the consensus panel of organisms.

In order to select the 34 human sequences in addition to the reference sequence, we decided upon a set of haplogroups such that no one population (e.g. European haplogroups) was over-represented. Example sequences from major haplogroups were then selected to ensure that all global regions were represented, using Phylotree (http://www.phylotree.org/) (van Oven and Kayser, 2009). Multiple sequence alignments were performed on mt-tRNA gene sequences from each species using ClustalW2 (http://www.ebi.ac.uk/Tools/msa/clustalw2/) (Larkin et al., 2007). Graphs were generated using GraphPad Prism (www.graphpad.com) software.

## 3 Results

### 3.1 Frequency of species reporting

**Fig. 1:**
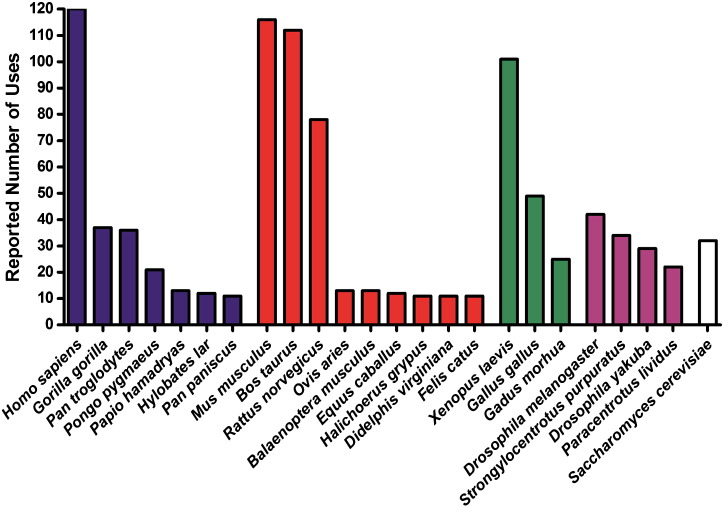
*Species utilisation habits when assessing evolutionary conservation*. This graph shows the number of times each species has been used to determine degree of conservation (excluding those species used fewer than 10 times). The data has been split into different shaded groups (from left to right): primates (black), other mammals (red), other vertebrates (green), other metazoans (purple), other eukaryotes (white).

Whilst a degree of variation in those species selected for determining conservation might be expected, the extent of this variability is alarming. A total of 163 different eukaryotic organisms (and 11 prokaryotes/viruses) were reported across the 120 papers. Of these 163 species, 68 were reported more than once, 46 were reported 5 times or more and 24 more than 10 times (Fig. 1). Interestingly, only 56% (67) of the 120 papers included any primate species other than *H. sapiens*. As might be expected, model organisms including *M. musculus*, *R. norvegicus*, *X. laevis*, *D. melanogaster* and *S. cerevisiae* were generally reported more often than other organisms.

### 3.2 Assessing intra-species variation

**Fig. 2:**
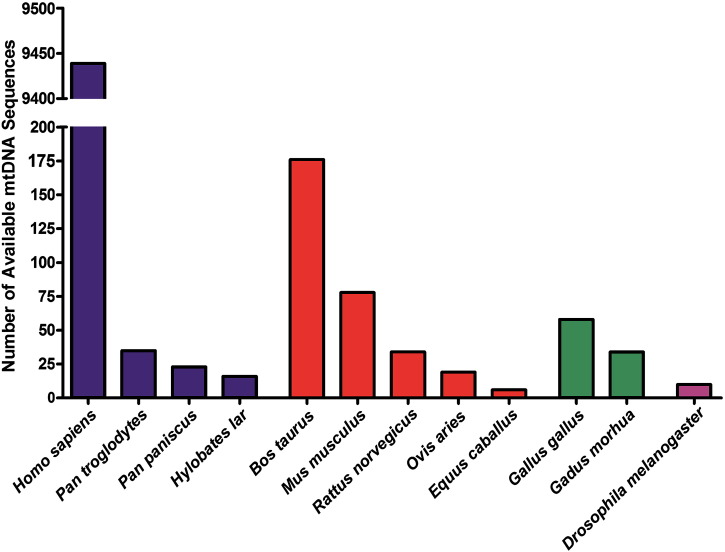
*Availability of mtDNA sequences in GenBank*®. This graph shows how many mtDNA sequences from each species are available in *GenBank*® (excluding species used 10 times or less and those with fewer than 5 available sequences) as of October 10th 2011. The data has been split into different coloured groups (from left to right): primates (blue), other mammals (red), other vertebrates (green) and other metazoans (purple).

The number of complete and nearly complete mtDNA sequences that were selected from GenBank® for each of the 24 species shown in Fig. 1 was calculated and displayed in Fig. 2, if multiple sequences (more than 5) were available. Only 50% (12) of the species used 10 times or more had 5 or more sequences available in GenBank®, and all these sequences are complete mtDNA genomes except for 32 *G. morhua* sequences (which are missing mt-tRNA^Pro^) and 6 *D. melanogaster* sequences (missing a number of mt-tRNAs).

**Supplementary Fig. 2:**
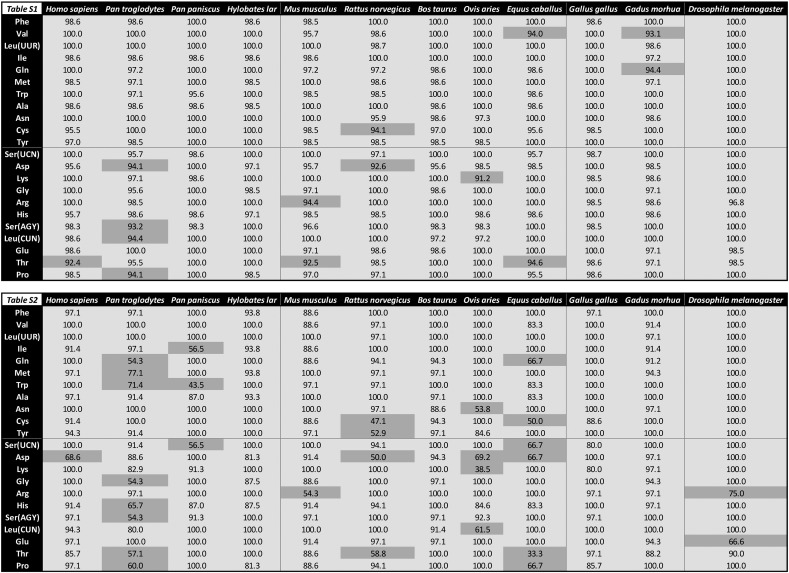
Raw mt-tRNA conservation data corresponding to Fig. 3. Table S1 shows the raw data linked to Fig. 3A, and is shaded such that those mt-tRNAs with ≥ 95% base conservation are light grey and those with < 95% conservation are dark grey. Table S2 shows the raw data linked to Fig. 3B, and is shaded such that those mt-tRNAs with ≥ 80% of sequences forming one identically conserved group are light grey and those mt-tRNAs with < 80% of sequences forming one identically conserved group are dark grey.

**Fig. 3:**
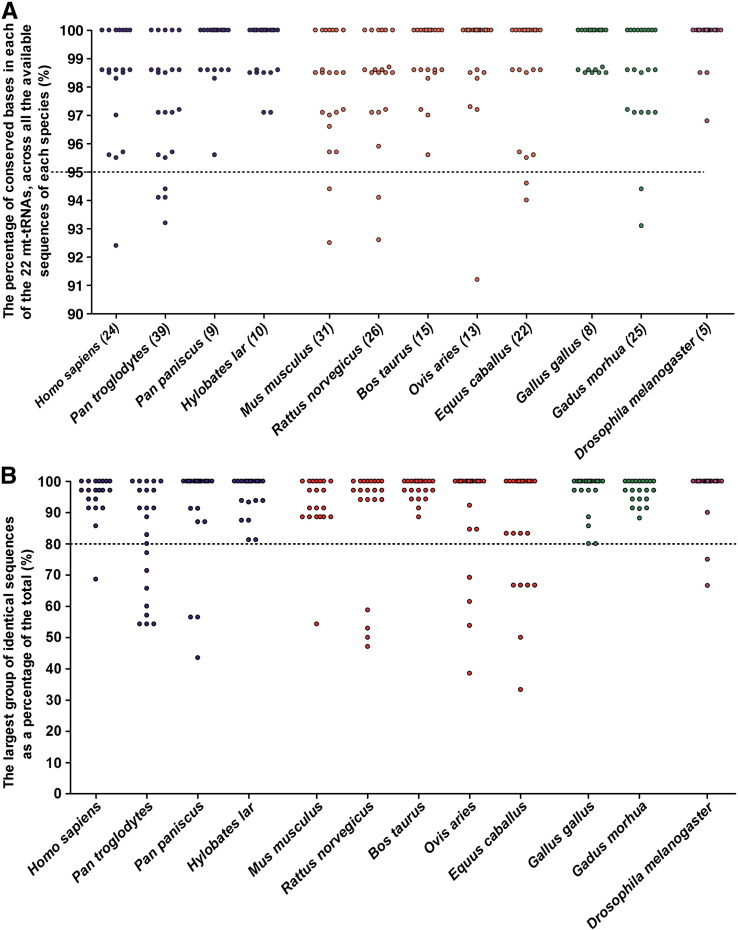
*Conservation of the mt-tRNAs*. A) This graph shows the percentage of bases within each mt-tRNA that are completely conserved across each sequence analysed for each species. Each mt-tRNA is represented by a dot and the total number of bases showing variation across the 22 mt-tRNAs in each species is displayed in the x-axis. B) Each point represents the percentage of identical sequences that make up the largest group for each mt-tRNA. The mt-tRNA identity of each individual point on either graph can be determined using Supplementary Fig. 2. In both Section A and Section B, the datda is split into different coloured groups (from left to right): primates (blue), other mammals (red), other vertebrates (green) and other metazonas (purple). *Conservation of the mt-tRNAs*. A) This graph shows the percentage of bases within each mt-tRNA that are completely conserved across each sequence analysed for each species. Each mt-tRNA is represented by a dot and the total number of bases showing variation across the 22 mt-tRNAs in each species is displayed in the x-axis. B) Each point represents the percentage of identical sequences that make up the largest group for each mt-tRNA. The mt-tRNA identity of each individual point on either graph can be determined using Supplementary Fig. 2. In both Section A and Section B, the datda is split into different coloured groups (from left to right): primates (blue), other mammals (red), other vertebrates (green) and other metazonas (purple).

There is a higher than expected level of intra-species conservation for almost all the 22 mt-tRNAs in every one of the species analysed. As many as 250 (95%) of the 264 mt-tRNAs show less than 5% base variation between analysed sequences within a species (Fig. 3A); over 80% of analysed sequences fall into one main identically conserved group in 235 (89%) of the mt-tRNAs (Fig. 3B). For 90% of the 264 mt-tRNAs, the reference sequence allele was the major allele, indicating that it was a good representation of the species. The individual mt-tRNAs (represented by the open circles in Fig. 3) can be identified using the raw data shown in Supplementary Fig. 2.

When variation is considered across all 12 species, just 229 bases out of an approximately 18,096 possible bases show any changes (1.3%). Interestingly, a Fisher's Exact Test showed that there is a significant difference (P < 0.0001), between the expected number of variations in the stems (139) and the loops (68), and the observed number of variations in the stems (78) and the loops (131) across the 12 species, with the T loop being particularly variable (85). Whilst most of the 22 mt-tRNAs cumulatively contain between 2.2% (5) and 6.2% (14) of the total variations seen, there are three exceptions. Mt-tRNA^Asp^ (10.1%) and mt-tRNA^Thr^ (9.7%) both show much higher levels of variation, whilst mt-tRNA^LeuUUR^ (0.9%) shows greater conservation.

### 3.3 The conservation panel

The conservation consensus panel will be used for assessing the pathogenicity of human mt-tRNA mutations and therefore benefits from being weighted towards primates, whilst maintaining sufficient species diversity to be representative of taxonomic evolution. In order to achieve this given that the 12 species that fulfil both selection criteria were mammal-orientated, we decided to include only 3 of the 5 suitable mammals. Both *M. musculus* and *R. norvegicus* are model organisms and therefore well studied, hence their inclusion in the panel. Finally, *B. taurus* was chosen for inclusion ahead of both *E. caballus* and *O. aries*, due to the availability of considerably more sequence data. The remaining 10 species therefore make up the proposed consensus panel of species (Table 1), and use the provisional or confirmed “reference sequence” as described in GenBank® (Accession numbers provided in Table 1).

## 4 Discussion

Assessing pathogenicity of a novel substitution in an mt-tRNA gene involves evaluation of the evolutionary conservation of the base involved. However, the uniformity of approach to assessing pathogenicity has not been matched by an equally consistent species selection. Conservation of the base suggests evolutionary importance and therefore change might be anticipated to have pathological consequences. Subjective species selection can imply conservation of a base where this does not exist and if other strands of evidence are supportive then pathogenicity can be erroneously misappropriated. To correct this, it will be necessary to consistently apply the same criteria to each mt-tRNA mutation being assessed, and a consensus panel of species by which to gauge evolutionary conservation would significantly improve consistency.

To assist in producing a concise and balanced consensus panel that is also consistent with previous reporting habits, two selection criteria were applied in order to narrow down the list of species considered for inclusion. Firstly, species must have been used to determine conservation 10 times or more in the published literature. This excluded species that are rarely used and ensured comparability with previous reports. Secondly, more than 5 mtDNA genomes must have been available in GenBank® to enable multiple sequence alignment analysis of the mt-tRNA genes. This allowed an assessment of both intra-species variability, which confirms the suitability of using one sequence to represent a species, and inter-species variability which supports the overall reliability and usefulness of a consensus panel of organisms for evolutionary conservation.

We have demonstrated the very large range of organisms (163 up to 31st September 2011) that have been used by the 120 reports of mt-tRNA mutations when determining evolutionary conservation. Whilst the majority of reports used species from across the eukaryotic background, others tended to focus on mammalian species. Oddly, given the use of conservation panels as a means of assessing the pathogenicity of human mt-tRNA variants, as many as 67 papers reported a range of species that did not include any primates other than *H. sapiens*. In addition to variability in species selection, there is also considerable variability in the number of species selected. Panels range in size from 2 to 86 species (not including whole databases), and such variation adds to the unreliability of comparative evolutionary conservation assessment. It is clear therefore, that constructing a standardised consensus panel of species (both in terms of size and species selected) on the basis of evolutionary relationships will be of great value.

Consistent with previous observations regarding the distribution of pathogenic mutations and neutral polymorphisms, variations were found to be more prevalent in mt-tRNA loops than stems. Variation hotspots in particular mt-tRNAs also confirmed that previously observed links between affected mt-tRNA and variant pathogenicity are not an artefact of targeted mtDNA sequencing. Assessing intra-species diversity and evolutionary patterns has often been performed using only non-coding control (D-loop) or *MTCYTB* (cytochrome *b* gene) mtDNA sequences for organisms other than humans (Thinh et al., 2010). However in recent years, there has been a realisation that using such small sections of the mtDNA is not sufficiently reliable for determining phylogeny (Chan et al., 2010). Consequently, a number of studies have recently been published that have sequenced the whole mtDNA genomes from a number of individuals within a species, allowing more accurate phylogenetic analysis (Bjork et al., 2011; Zsurka et al., 2010). All published mtDNA sequences are available in GenBank®, and we found there a remarkably low level of intra-species diversity for each mt-tRNA within each of the species investigated (including *H. sapiens*).

Sequence analysis using multiple alignments has demonstrated the validity of building a consensus panel of organisms using one reference sequence to represent each species. The majority of mt-tRNAs in each species have one major group of identical sequences, whilst only a few contain two large groups that differ by one or two bases. This is likely to be indicative of the presence of haplogroup-defining variations. For instance, 68% of human mt-tRNA^Asp^ sequences have a G nucleotide at position 7521, whilst 32% have an A nucleotide. This position is a known SNP associated with a number of specific haplogroups (van Oven and Kayser, 2009). The GenBank®-denoted “reference sequence” was not always the optimum match for each mt-tRNA, most likely as a consequence of the fact that reference sequences are often simply the first sequence completed. In the majority of cases however, any variation between groups of sequences tends to be minimal and for ease of use, the provisional or confirmed “reference sequence” for each species should be used. With an increasing number of organisms being sequenced, assigned reference sequences may be altered in the future. This work demonstrates that changing the representative sequence of a species in the consensus panel would be valid, and the panel's usefulness would be maintained.

### 4.1 The conservation panel

The consensus panel proposed here is biased towards primates, with a bonobo (*P. paniscus*), a common chimpanzee (*P. troglodytes*) and a gibbon (*H. lar*) included along with *H. sapiens*. Also included are 3 further mammalian species, including the two model organisms *R. norvegicus* (rat) and *M. musculus* (mouse) as well as *B. taurus* (cow). The inclusion of a fish (*G. morhua* (Atlantic cod)) a bird (*G. gallus* (chicken)) species and the fruit fly (*D. melanogaster*: a distant, model invertebrate organism) ensures that evolutionary conservation is being assessed across a diverse range of species.

It is worth noting that despite the popularity of using both *X. laevis* and *S. cerevisiae* (two model organisms) for assessing conservation (see Fig. 1), neither has been included in our panel due to the lack of sequence data available in GenBank®. The absence of these species from the final panel does not greatly affect the diversity, given the size of the panel and the range of non-mammalian species selected, but it is possible that a future revision of this panel may result in their inclusion. Although a larger panel of species would enable greater taxonomic diversity to be assessed, the benefits of including more species do not outweigh the benefits of a streamlined user-friendly panel. Given also, that many panels used in the literature consist of just 3 or 4 species, this panel is already somewhat larger and more diverse than those commonly used to date.

### 4.2 Scoring evolutionary conservation

**Fig. 4:**
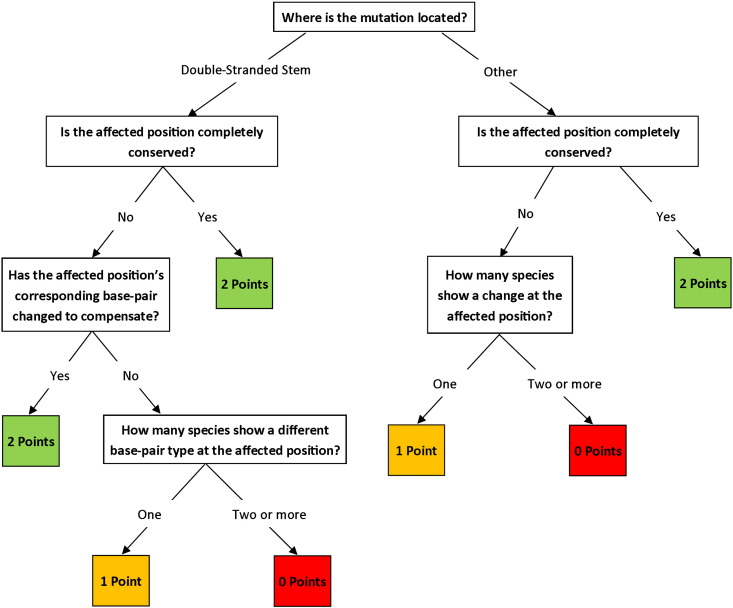
*Flowchart for scoring conservation*. The region of the mt-tRNA in which a mutation occurs influences how pathogenicity should be scored. If not located in a double-stranded stem, then the only consideration is how many changes are seen at this position across the panel of species. If however, the mutation is in a double-stranded stem, then whilst the position itself should be considered first, consideration should also be made for the base-pairing (i.e. Watson–Crick or not) in which it is involved. Scores should be calculated according to this flowchart green boxes score 2 points, yellow boxes one point and red boxes 0 points and included in the final pathogenicity score outlined by our pathogenicity scoring system (Yarham et al., 2011).

To clarify the way in which conservation should be scored within the constraints of our pathogenicity scoring system (Yarham et al., 2011), we have developed the following flowchart (Fig. 4). One inevitable issue that arises from any assessment of conservation in genetic sequences is that post-transcriptional modifications cannot be considered, meaning that the analysis is performed on “raw” genetic data. However, the conservation status of an affected base is not and should never be the only requirement for determining pathogenicity of mt-tRNA variants. It is simply one facet of the mt-tRNA point mutation pathogenicity scoring system outlined previously (Yarham et al., 2011).

The conservation score assigned by our pathogenicity scoring system for most of the reported mutations remains the same after application of this consensus panel of species (e.g. m.3243A>G (Goto et al., 1990) and m.4269A>G (Taniike et al., 1992) score 2 points, and m.8344A>G (Shoffner et al., 1990) scores 0 points), many obtain a lower score (e.g. m.602C>T (Sakiyama et al., 2011) decreases by 2 points, whilst m.4285T>C (Silvestri et al., 1996) and m.7510T>C (Hutchin et al., 2000) decrease by 1 point) and a few receive a higher score (e.g. 608A>G (Tzen et al., 2001) gains 2 points). The examples described here are not the only reported mutations to be affected by this change in conservation panel, but they illustrate the benefits consistency will provide.

## 5 Conclusions

This work shows that greater consistency in species selection is crucial for maximising the usefulness of conservation as a measure of pathogenicity. The reliability and usefulness of a panel of organisms represented by a single sequence are demonstrated, and the value of this consensus panel in improving determination of pathogenicity of mt-tRNA variants as part of the pathogenicity scoring system is shown.

The following are the supplementary materials related to this article.

## Figures and Tables

**Table 1:** Proposed consensus panel of organisms. This table lists the 10 species that we propose should be consistently used as part of a consensus panel to determine the degree of conservation of mutated mt-tRNA bases. The GenBank® accession number for each species' reference sequence is also provided.

Taxonomic classification	Species	GenBank® reference sequence accession number
Primates	*Homo sapiens*	NC_012920
	*Pan troglodytes*	NC_001643
	*Pan paniscus*	NC_001644
	*Hylobates lar*	NC_002081
Mammals	*Mus musculus*	NC_005089
	*Rattus norvegicus*	NC_001665.2
	*Bos taurus*	NC_006853
Vertebrates	*Gallus gallus*	NC_001323
	*Gadus morhua*	NC_002081
Invertebrates	*Drosophila melanogaster*	NC_001709
